# Subjective wellbeing among rheumatic heart disease patients at Tikur Anbessa Specialized Hospital, Addis Ababa, Ethiopia: observational cross-sectional study

**DOI:** 10.1186/s12913-021-07378-0

**Published:** 2021-12-19

**Authors:** Henok Tadele, Hayat Ahmed, Helen Mintesnot, Etsegenet Gedlu, Senbeta Guteta, Dejuma Yadeta

**Affiliations:** 1 Department of Pediatrics and Child Health, Cardiology Division, School of Medicine, College of Health Sciences, Addis Ababa University, Addis Ababa, Ethiopia; 2 Department of Internal Medicine, Cardiology Division, School of Medicine, College of Health Sciences, Addis Ababa University, Addis Ababa, Ethiopia

**Keywords:** Subjective wellbeing, Rheumatic heart disease, Depression, Ethiopia

## Abstract

**Background:**

Subjective wellbeing (SWB) is a self-reported positive life judgment and good feeling. RHD, rheumatic heart disease, is a long-term sequel of single or recurrent acute rheumatic fever. There are no studies that assessed SWB in RHD patients. We aimed to assess SWB among RHD subjects enrolled in chronic care at Tikur Anbessa Specialized Hospital (TASH), Ethiopia.

**Methods:**

This observational cross-sectional study employed a validated Amharic WHO-5 wellbeing index to assess SWB. Sociodemographic and clinical data were collected using structured questionnaire. RHD subjects aged 9 years and above were included. Factors associated with SWB were assessed using logistic regression models.

**Results:**

The study included 384 subjects, females 68.2% (262). Children, < 18 years, constituted one third of study subjects, 32.8% (126). Moderate and severe echocardiographic RHD dominated, 85.9% (330) with no associated comorbidity, 84.4% (324). Only 17.2% (66) had surgical or device intervention. Poor SWB was documented in 9.6% of study subjects (95% CI: 6.88–13.04). On multivariable regression, those with younger age RHD diagnosis, < 20 years, had almost three times higher odds of poor SWB, adjusted odds ratio (aOR) 2.69(95% CI: 1.30–5.58, P 0.008). Those with monthly family income of < 1000 Ethiopian Birr had three times higher odds of poor SWB, aOR 2.97(95% CI: 1.24–7.1, P 0.014). Study subjects who had good medication adherence had reduced odds of poor SWB, aOR 0.37(95% CI: 0.18–0.77, P 0.028). Those who received psychologic support from their families also had reduced odds of poor SWB, aOR 0.26(95% CI: 0.11–0.64, P 0.003).

**Conclusion:**

Poor SWB was documented in one-tenth of RHD patients. Family income, younger age at RHD diagnosis, medication adherence and psychological support predicted poor SWB. Poor SWB has to be considered and assessed among RHD patients particularly among those with younger age at RHD diagnosis and poor family income. Further mixed studies are recommended to assess how medication adherence and psychological supports associate with positive SWB among RHD patients.

**Supplementary Information:**

The online version contains supplementary material available at 10.1186/s12913-021-07378-0.

## Background

Health is defined by the World Health Organization (WHO) as a state of complete physical, mental and social wellbeing, and not merely an absence of disease [1]. Good health and wellbeing are included as the third Sustainable Development Goal (SDG) by the United Nations [2]. Wellbeing is defined as a self-reported positive life judgement and good feeling [3]. Subjective wellbeing (SWB) assesses affective and cognitive evaluation of a person’s life [4]. SWB is measured using self-report of affective and cognitive domains by different measures [3, 5]. While several measures were extensively studied in developed nations, a validated SWB assessment tool was not made available till 1998 when WHO introduced a new SWB measure for wider use at global scale. The WHO-5 wellbeing index uses a questionnaire that assesses the current mental wellbeing (in the last 2 weeks) of a subject. It contains 5 simple questions which were produced after several revisions during clinical trials. It was revised only to have positively phrased questions to increase study subject acceptance and to avoid negative symptom related issues [6]. A systematic review of the literatures was conducted to assess the validity of the WHO-5 wellbeing index for depression screening and outcome measures in several clinical trials, and it was reported as a valid screening tool [7].

Rheumatic heart disease (RHD) is the long-term cardiac sequelae caused by a single severe or recurrent acute rheumatic fever episodes [8]. RHD is believed to be the disease of the poor owing to its high prevalence in these low income settings in the face of effective preventive strategies [9]. Echocardiography based Ethiopian studies documented RHD to be a very common (highly endemic) among school children [10], and RHD was also reported as the top cardiac disease among cardiac patients receiving chronic care in the main referral centers [11]. A national guideline for treatment, prevention and control as well as RHD registries was published by the Ethiopian Ministry of Health [12]. Seven action points for RHD eradication in Africa were forwarded using the Addis Ababa communiqué. These action points include establishment of RHD registries, ensuring benzathine penicillin availability, access to universal reproductive health services for women with RHD, decentralizing the RHD services to districts, increasing cardiac surgery centres, multisectoral collaboration, and cultivating partnership to ensure the application of the action points [13]. The African Union also took an initiative to embark on RHD eradication from Africa [14].

Studies have documented that RHD patients suffer from a great deal of depression and anxiety [15]. An international study on a review of the world mental health surveys concluded mental disorders particularly anxiety-mood disorders as common problems among heart disease patients [16]. High level of cardiac disease anxiety was postulated to be the predisposing factor for depression [15, 16].

Positive wellbeing is considered to be protective for cardiovascular diseases/events and grants long life. Proposed mechanisms are interrelated including adaptive physiologic functioning, promotion of positive health behaviors, and buffering the effect of stress on health. Moreover, good health would also bring positive wellbeing [17–19]. Wellbeing index has been shown to increase after cardiac rehabilitation among ischemic heart disease and obese patients [20]. Compared to other screening tools, the WHO- 5 wellbeing index has been shown to be a reliable SWB screening tool among patients with chronic illnesses [21]. Definitive surgical or percutaneous treatments for RHD are not readily available in Ethiopia and hence patients live with severe manifestations and complications of RHD affecting their quality of life [22]. There are no studies that assessed the SWB in RHD patients. Hence, we aimed to assess SWB and its determinants among RHD patients enrolled on chronic care at the only tertiary national cardiac referral center for Ethiopia.

## Methods

### Study area

The study was conducted in Addis Ababa, Ethiopia at Tikur Anbessa Specialized Hospital (TASH). It is the teaching hospital of Addis Ababa University, College of Health Sciences, and School of Medicine. It is also the largest referral hospital in Ethiopia with multiple specialties and sub-specialties. It is the national referral cardiac center. There are over 5000 RHD patients aged 9 years and above enrolled in chronic care and follow up. Pediatrics and adult cardiac clinics are attended by consultant pediatric / adult cardiologists, pediatric/ adult cardiology fellows, pediatric/ internal medicine residents and trained nurses.

### Study design and study period

The study employed cross-sectional study design and it was conducted between December 2020 and May 2021.

### Study population

The study population was all children above the age of 9 years and adults with RHD attending follow up at the pediatric and adult cardiology clinics.

### Sample size and sampling procedure

A sample size of 384 was calculated using assumptions of 50% poor SWB among RHD patients, 95% confidence level with 5% margin of error and a power of 80. A proportion of 50% poor SWB was used as there are no previous studies on SWB among RHD patients. There was an existing RHD registry in both adult and Pediatric Cardiology clinics and it was used as sampling frame. Subjects aged 9 years and above were included, and simple random sampling technique (manual lottery method) was used to identify or select study subjects by the data collectors as they show up for follow up. The pediatric to adult cardiac patients’ sample selection was made using proportional to sample size allocation.

### Inclusion and exclusion criteria

All RHD patients aged 9 years and above with established echocardiographic diagnosis and who were on follow up for over 6 months were included in the study. Study subjects with acute illness like heart failure and subjects with previously diagnosed or acute psychiatric illness were excluded from the study as they required immediate medical attention and care.

### Data collection

Demographic and clinical data were collected using structured questionnaire prepared for the purpose of this study and pretested on non-participating patients. Subjective Well-being (SWB) was assessed using a validated Amharic translated WHO- 5 wellbeing tool [23]. The tool is approved for use among children above 9 years of age, adolescents as well as adults. It has 5 statements on how patients have been feeling over a period of 2 weeks before the time of the study. A scale of 0–5 is provided in descending order to rate the amount of time the feelings lasted. Higher numbers indicate better subjective well-being. The raw score is calculated by totaling the figures of the five answers. The raw score ranges from 0 to 25, 0 representing worst possible and 25 representing best possible quality of life. A raw score below 13 indicates poor wellbeing and is an indication for assessment of depression. Clinical and sociodemographic data were collected by BSc Nurses who received a one-day training on the tools. The quality of collected data was checked daily by investigators.

### Variables

The dependent variable was subjective well-being score. Independent variables include age, gender, address, distance from the health facility/TASH, education, marital status, number of children, family income, occupation, age at RHD diagnosis, New York Heart Association (NYHA) functional class, severity of RHD, any comorbidity, comorbid chronic illness, RHD related complication, number of drugs, medication adherence, history of cardiac intervention, psychological support, hospital admission in the last 1 year, and nutritional status.

### Operational definitions

Rheumatic heart disease was defined as damage to the heart, particularly the heart valves, after having one or more attacks of rheumatic fever [8]. NYHA functional status was functional classification of patients according to cardiac functional capacity [24]. Subjective well-being was defined as a self-reported positive life judgment and good feeling [3]. WHO subjective well-being index is a questionnaire that assesses the current mental wellbeing in the last 2 weeks of a subject using five questions [6]. Rheumatic heart disease was classified as mild, moderate and severe based on the World Heart Federation Criteria as assessed by echocardiography [25]. Medication adherence was assessed based on patient self-report. Good adherence was considered when patient had taken > 80% of prescribed medications before clinic visit date while satisfactory adherence was considered when between 60 and 80% of medications were taken. Poor adherence was considered when < 60% of prescribed medications were taken. Psychological support was defined as any form of support provided to the patient in terms of reminder of follow up visits, pill time reminders, accompany during ill periods or clinic visits, and provision of hope and encouragement.

### Data analysis

Data completeness was checked manually and entered into Statistical Software for Social Sciences (SPSS) version 25 for analysis. Chi-square analysis was conducted to check for univariate association. Then, variables with *p* value less than 0.05 were selected from binary regression for final multivariable logistic regression analysis. Association between dependent and independent variables was measured using odds ratio and 95% confidence intervals. *P* value less than 0.05 was set as statistically significant.

## Results

### Sociodemographic characteristics and SWB scores of study subjects

A total of 402 study subjects were approached, and 18 were excluded (8 cases of heart failure requiring admitted care, 2 cases of acute or previous psychiatric illness, and 8 cases of non-consent). And, finally this study included 384 study subjects with female predominance, 68.2% (262). The mean age of study subjects was 27.03 + 12.85 years. Children below the age of 18 years constituted 32.8% (126) of study subjects. Majority of study subjects were from Addis Ababa, 49.7% (191) followed by Oromia region, 32.3% (124). Majority of study subjects lived within 100-km from the study health facility (TASH) or Addis Ababa, 56.5% (217). Majority of them also had completed primary education and above, 86.6% (333).

Concerning marital status, majority of study subjects were single, 57.3% (220). One hundred fifty-six (40.6%) of study subjects were students. Almost 80 % of study subjects had a monthly family income of 1000 and above Ethiopian birr. There was no difference in SWB between children and adults. No association was noted with sex, residence and distance from health facility, occupation, and SWB. Only family income was associated with SWB from sociodemographic variables (See Table 1).

**Table 1:** Sociodemographic characteristics and Chi-square analysis outputs of Rheumatic Heart Disease study subjects at Tikur Anbessa Specialized Hospital, 2021

Variable	Frequency(n)	Percentage (%)	SWB: n (%), *P* value		
			Poor	Normal	***P*** value
Age (yrs)					0.248
< 18	126	32.8	9(7.1)	117(92.9)	
> 18	258	67.2	28(10.3)	230(89.1)	
Sex					0.516
Male	122	31.8	10(8.2)	112(91.8)	
Female	262	68.2	27(10.3)	235(89.7)	
Address: Regions					0.631
Addis Ababa	191	49.7	22(11.5)	169(88.5)	
Oromia	124	32.3	11(8.9)	113(91.1)	
Amhara	36	9.4	3(8.3)	33(91.7)	
SNNPR	32	8.3	1(3.1)	31(96.9)	
Others	1	0.3	0(0)	1(100)	
Distance from follow up health facility (in kilometers)					0.281
< 100	217	56.5	26(12)	191(88)	
100–199	47	12.2	3(6.4)	44(93.6)	
200–299	55	14.3	5(9.1)	50(90.9)	
> 300	65	16.9	3(4.6)	62(95.4)	
Educational Status					0.159
Illiterate	40	10.4	7(17.5)	33(82.5)	
Can read and write	11	2.9	1(9.1)	10(90.9)	
Primary education (1–8)	148	38.5	8(5.4)	140(94.6)	
Secondary education (9–12)	118	30.7	14(11.9)	104(88.1)	
College level (Certificate/diploma/first degree/MS/MD/PHD)	67	17.4	7(10.4)	60(89.6)	
Marital status					0.426
Married	153	39.8	17(11.1)	136(88.9)	
Single, divorced, separated or widow	231	60.2	20(8.7)	211(91.3)	
Number of your children					0.216
One	42	10.9	5(11.9)	37(88.1)	
Two	59	15.4	10(16.9)	49(83.1)	
Three	21	5.5	2(9.5)	19(90.5)	
Four and above	30	7.8	1(3.3)	29(96.7)	
Occupation					0.441
Student	156	40.6	11(7.1)	145(92.9)	
Government employee	40	10.4	3(7.5)	37(92.5)	
Self-employed	71	18.5	10(14.1)	61(85.9)	
Housewife	92	24	9(9.8)	83(90.2)	
Others (Farmer, daily laborer)	25	6.5	4(16)	21(84)	
Family monthly income (Ethiopian Birr)					0.008
< 1000	80	20.8	15(18.8)	65(81.3)	
1000–2999	155	40.4	11(7.1)	144(92.9)	
> 3000	149	38.8	11(7.4)	138(92.6)	

### Clinical characteristics and SWB scores of study subjects

The mean age at RHD diagnosis was 17.14 + 10.78 years. Majority of our study subjects had moderate to severe RHD as assessed by echocardiography, 85.9% (330). Most of our study subjects didn’t report any associated comorbidity, 84.4% (324). Seizure disorders, hypertension and HIV were the common reported comorbidities.

Most of our study subjects were in NYHA class I and II during the time of assessment, 62.5% (240). Heart failure, 12.5% (48), was the common reported RHD complication. Stroke and rheumatic recurrence occurred in 4.9% (19) and 4.7% (18) of study subjects, respectively. Almost all of our study subjects were on multiple medications for RHD, 97.7% (375) and majority reported good medication adherence, 77.4% (297).

Most of study subjects received psychologic support from their families, 90.9% (349). A quarter of them had hospital admission in the last 1 year, 26% (100). A little over one- tenth of study subjects had surgical intervention for their RHD, 17.2% (66). Majority of study subjects had normal anthropometric indices, 87.8% (337). Age at RHD diagnosis, medication adherence and psychological support showed association with SWB. However, RHD severity, NYHA class, comorbidities, RHD related complications, number of medications taken, RHD intervention, hospital admission in the last 1 year, and nutritional status didn’t show any association with SWB (See Table 2).

**Table 2:** Clinical characteristics and Chi-square analysis outputs of Rheumatic Heart Disease study subjects at Tikur Anbessa Specialized Hospital, 2021

Variable	Frequency(n)	Percentage- (%)	SWB: n (%), *P* value		
			Poor	Normal	*P* Value
Age at RHD diagnosis(yrs)					0.005
< 20	264	68.8	18(6.8)	246(93.2)	
20 and above	120	31.2	19(15.8)	101(84.2)	
RHD severity					0.456
Mild	54	14.1	4(7.4)	50(92.6)	
Moderate	86	22.4	6(7)	80(93)	
Severe	244	63.5	27(11.1)	217(88.9)	
Any comorbidity					0.126
No	324	84.4	28(8.6)	296(91.4)	
Yes	60	15.6	9(15)	51(85)	
Common Comorbidities or conditions					0.120
Seizure disorders	7	1.8	1(14.3)	6(85.7)	
Hypertension	5	1.3	1(20)	4(80)	
HIV	4	1.0	0(0)	4(100)	
Chorea	4	1.0	1(25)	3(75)	
Pregnancy	2	0.5	0(0)	2(100)	
Others (anemia, myoma, pituitary adenoma, hypothyroidism etc)	24	6.3	4(16.7)	20(83.3)	
NYHA class					0.366
I	76	19.8	5(6.6)	71(93.4)	
II	164	42.7	15(9.1)	149(90.9)	
III	50	13	8(16)	42(84)	
IV	94	24.5	9(9.6)	85(90.4)	
RHD related complication					0.360
HF	48	12.5	4(8.3)	44(91.7)	
Stroke	16	4.2	0(0)	16(100)	
Rheumatic Recurrence	18	4.7	0(0)	18(100)	
Others (AF, AV block, LA thrombus…)	46	12	8(17.4)	38(82.6)	
Number of drugs					0.880
Single	9	2.3	1(11.1)	8(88.9)	
Multiple	375	97.7	36(9.6)	339(90.4)	
Adherence to medications					0.021
Good	297	77.3	23(7.7)	274(92.3)	
Poor or Satisfactory	87	22.7	14(16.1)	73(83.9)	
Psychological support obtained from					0.000
Family	349	90.9	27(7.7)	322(92.3)	
Friends	35	9.1	10(28.6)	25(71.4)	
Hospital admission in the last 1 year					0.086
Yes	100	26	14(14)	86(86)	
No	284	74	23(8.1)	261(91.9)	
RHD intervention (Surgical or device)					0.770
Yes	66	17.2	7(10.6)	59(89.4)	
No	318	82.8	30(9.4)	288(90.6)	
Nutritional status					0.205
Normal	337	87.8	31(9.2)	306(90.8)	
Mild wasting	29	7.6	4(13.8)	25(86.2)	
Moderate wasting	16	4.2	1(6.3)	15(93.8)	
Severe wasting	2	0.5	1(50)	1(50)	

### Subjective wellbeing score

Poor SWB was reported in 9.6% (95% CI: 6.88–13.04) of study subjects. Worst SWB of 0 or feeling at no time was commonly reported for a question that asks ‘daily life is filled with things that interest me’, 5.2% (20). Response of sometimes was commonly reported for a question that asks ‘I have felt active and vigorous’, 5.7% (22). The best SWB response of all of the time was documented for the question that asks ‘I woke up feeling fresh and rested’, 50.8% (95) (See Table 3).

**Table 3:** Subjective wellbeing score of rheumatic heart disease patients at Tikur Anbessa Hospital based on WHO 5 wellbeing score, 2021

Variable	n (%)					
SWB score						
13 or above	347(90.4)					
Below 13	37(9.6)					
SWB individual Scores	**Score, n (%)**					
	0	1	2	3	4	5
WHO1	10(2.6)	19(4.9)	15(3.9)	46(12)	120(31.3)	174(45.3)
WHO2	9(2.3)	19(4.9)	10(2.6)	57(14.8)	122(31.8)	167(43.5)
WHO3	8(2.1)	22(5.7)	15(3.9)	57(14.8)	112(29.2)	170(44.3)
WHO4	9(2.3)	19(4.9)	9(2.3)	47(12.2)	105(27.3)	195(50.8)
WHO5	20(5.2)	15(3.9)	20(5.2)	63(16.4)	115(29.9)	151(39.3)

### Factors associated with SWB among RHD study subjects

Study subjects with family income of < 1000ETB per month had three times higher odds of poor SWB, aOR 2.97(95%: 1.24–7.1, P 0.014). Those with younger age RHD diagnosis, < 20 years, had almost three times higher odds of poor SWB, aOR 2.69(95% CI: 1.30–5.58, P 0.008). Study subjects who had good medication adherence had reduced odds of poor SWB, aOR 0.37(95% CI: 0.18–0.77, P 0.028). Those who received psychologic support from their families also had reduced odds of poor SWB, aOR 0.26(95% CI: 0.11–0.64, P 0.003) (See Table 4).

**Table 4:** Multivariable logistic regression output of factors associated with poor SWB among patients with Rheumatic Heart Disease at Tikur Anbessa Specialized Hospital, 2021

Variable	Categories	SWB		COR, *P* value	AOR *P* value	
		Poor SWB	Normal			
Family income						
	< 1000	15	65	2.89(1.26–6.65), P 0.012	2.97(1.24–7.1)	0.014
	1000–2999	11	144	0.96(0.40–2.2), P 0.923	0.9(0.37–2.2)	0.082
	> 3000	11	138	1	1	
Age at RHD diagnosis						
	< 20	18	246	0.39(0.19–0.77), P 0.007	2.69(1.30–5.58)	0.008
	> 20	19	101	1	1	
Adherence						
	Good	23	274	0.44(0.22–0.89), P 0.023	0.37(0.18–0.77)	0.028
	Poor/Satisfactory	14	73	1	1	
Psychologic support						
	Family	27	322	0.21(0.09–0.48), P 0.000	0.26(0.11–0.64)	0.003
	Others	10	25	1	1	

## Discussion

Our Study is the first to report SWB and its determinants among RHD patients from a large study sample. Unmarried females with RHD diagnosis at second decade of life predominated the study population. Children constituted one third of study subjects. Majority had moderate to severe RHD disease severity on echocardiography with no associated comorbidity. Most of our study subjects had mild to moderate clinical symptoms during the study and lived within 100-km from the follow up health facility. Heart failure was the most common reported RHD complication. Majority were on multiple medications with good adherence. Families provided psychologic support in most cases. A quarter had hospital admission in the last 1 year. Only a little over one tenth of them had surgical intervention for RHD. Poor SWB was reported in one tenth of RHD patients. Family income, age at RHD diagnosis, medication adherence and psychological support predicted poor SWB among RHD subjects.

Majority of our study subjects were females. Previous echocardiography based Ethiopian RHD studies documented consistently female predominance [10, 11, 26]. Higher autoimmunity with effect of sex hormones and better RHD detection during antenatal care in the reproductive age women were postulated reasons for higher RHD occurrence in females. More severe valvular lesions were also documented among females [27]. Social factor like child raising which increases ARF/RHD susceptibility are also incriminated as plausible explanation [28]. In our study, most of RHD subjects were not married. Ugandan study had documented presence of stigma and discrimination among patients with RHD [29]. Presence of cardiac symptoms and associated burdens could limit their desire for marriage. Detailed reasons for singled life among RHD need to be explored.

In this study, the mean age for RHD diagnosis was second decade of life. Moreover, children below the age of 18 years constituted a third of study population. This further reinforces the established evidence that RHD is the disease of children and young adults [30]. Ethiopia was also reported as a high endemic setting for RHD with children and young adults being most affected [10]. This underlines the need for strengthening the national RHD preventive strategies put in place to avert the national productivity loss [12].

In our study, majority of RHD subjects had moderate to severe valvular lesions on echocardiography. RHD disease progression in Sub-Saharan Africa was reported to be more accelerated [26, 31]. Lack of readily available definitive management could also make the case for such a severe disease distribution [22]. Our study subjects didn’t have major associated chronic illnesses. However, this should be interpreted in light of the younger of age of our study population where occurrence of chronic illnesses is said to be low.

In this study, heart failure, stroke and rheumatic recurrences were the most common documented RHD complications. Similar pattern was also reported in other studies [32–34]. Such a course is expected in the non-intervened RHD patients. Majority of RHD subjects were on multiple medications with good adherence. Good medication adherence was also reported in other studies [35, 36]. Our study subjects were already enrolled on chronic care, the medical care and clinical recommendations might have contributed to good medication adherence. However, the self-report of adherence could have inflated adherence responses.

In the current study, one tenth of RHD cases had poor SWB. There are no similar studies done on RHD to compare our figure. We believe this is significant given the burden of RHD in the nation and developing countries. Hence, it calls for timely consideration and prompt action.

In our study, family income less than 1000ETB was positively associated with poor SWB. The association between income and SWB is an area of debate among experts. High income was documented to be associated with poor SWB [37]. Weak or no association was reported in a meta-analysis [38]. Other studies documented positive association between income and well-being [39]. Further mixed qualitative and quantitative studies are recommended to explore this subject.

Younger age at RHD diagnosis, < 20 years, was positively associated with poor SWB. This is in agreement with the global report that documented reduced life satisfaction among adolescents [40]. Important life changes that included mood and life satisfaction, and expectation at school performance are known to affect SWB in adolescents [41]. Positive youth development through positive self-image, personal wellness, current or future aspirations and family connectedness were reported to associate with positive SWB among adolescents [42]. Presence of an established RHD with lack of access to definitive surgical interventions could put our study subjects at increased odds for poor SWB.

Good medication adherence was negatively associated with poor SWB in this study. Similar finding was reported in other chronic illness [43]. Good adherence was also reported to be associated with improved health outcomes and hence positive SWB [44]. Effects of cardiac medications on autonomic function could have contributed to good adherence and positive SWB. Reduced autonomic dysfunction that clinically translates to low heart rate variability, lower systolic blood pressure, and faster cardiovascular recovery was reported to be associated with positive SWB [45, 46]. Medication adherence could promote and associate to other healthy behaviors like healthy eating, physical activity, smoking cessation, and improved SWB [18, 47]. Further explorative mixed studies are needed to understand how medication adherence relates to SWB among RHD patients.

Psychological support from families was negatively associated with poor SWB in this study. Similar report was documented in another study [48]. Various assistances for chronically ill patients on chronic medications might help to improve their SWB [49]. However, absence of association was reported in another study. Their proposed explanation was that social support could provide comfort but might have negative effect on SWB or self-esteem [50]. While details are yet to be studied, Ethiopians have strong family and social supports schemes and that might have contributed for the strong family support and hence positive SWB.

The strengths of our study include inclusion of large sample from the majorly non-intervened RHD and use of validated SWB assessment tool, prospective data collection, and collection of social, demographic and clinical variables. Our study has some limitations. First, we have assessed medication adherence based on self-report and that has overrating risk. Second, we employed questionnaire-based study and we couldn’t exhaust assessment of predictors for SWB among the RHD as that will additionally require qualitative component for triangulation. Thirdly, we were not able to adjust for more variables due to sample of the study and small number of variables in final model. Lastly, our study analysis employed data mining approach owing to lack of evidence on determinants of SWB among RHD subjects. Future studies should consider the out puts of this study and other related literatures during analysis. However, this study provides insightful information on the quality of life among RHD patients and their associated risk for depression. It also helps policy makers to consider SWB assessment as a routine care in addition to the mandatory advancement of RHD interventions in our patients and in similar settings.

## Conclusion

In our study, one tenth of RHD subjects had poor SWB. Children and young adults with second decade of life RHD diagnosis predominated. Echocardiographically, most of them had moderate to severe RHD disease. Majority were in NYHA class I and II, and lived within 100-km from the follow up health facility. Heart failure was the most common reported RHD complication. Majority were on multiple medications with good adherence. Families provided psychologic support in most cases. Only a little over one tenth had surgical intervention for RHD. Family income, age at RHD diagnosis, medication adherence and psychological support predicted poor SWB among RHD subjects. Poor SWB has to be considered and assessed among RHD patients particularly among those with younger age at RHD diagnosis and poor family income. Further mixed studies are recommended to assess how medication adherence and psychological supports associate with positive SWB among RHD patients.

## Supplementary Information

**Additional file 1.**

**Additional file 2.**

## Data Availability

The datasets analyzed during this study are available from the corresponding author on reasonable request.

## Abbreviations

AF: Atrial Fibrillation
aOR: Adjusted odds ratio
AV block: Atrioventricular block
COR: Crude odds ratio
ETB: Ethiopian Birr
HF: Heart Failure
HIV: Human Immunodeficiency Virus
IE: Infective Endocarditis
LA: Left Atrium
MD: Medical doctor
MS: Masters of science
NYHA: New York Health Association
PhD: Doctorate of Philosophy
RHD: Rheumatic Heart Disease
RTA: Road Traffic Accident
SNNPR: Southern Nations and Nationalities Peoples Regional State
SPSS: Statistical Package for Social Sciences
SWB: Subjective Wellbeing
TASH: Tikur Anbessa Specialized Hospital
WHO: World Health Organization
