# Supplementary material for: Subjective wellbeing among rheumatic heart disease patients at Tikur Anbessa Specialized Hospital, Addis Ababa, Ethiopia: observational cross-sectional study
Source: BMC Health Serv Res. 2021 Dec 19;21:1354. doi: 10.1186/s12913-021-07378-0 (PMC8684619; doi:10.1186/s12913-021-07378-0)
Supplement: Supplementary file 1 — Additional file 1. [file 12913_2021_7378_MOESM1_ESM.docx]

**Psychiatric Research Unit** WHO Collaborating Centre in Mental Health


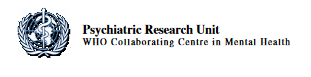


WHO (Five) Well-Being Index (1998 version)

Please indicate for each of the five statements which is closest to how you have been feeling over the last two weeks. Notice that higher numbers mean better well-being.

Example: If you have felt cheerful and in good spirits more than half of the time during the last two weeks, put a tick in the box with the number 3 in the upper right corner.

|  | *Over the last two weeks* | All of the time | Most of the time | More than half of the time | Less than  half of the time | Some of the time | At no time |
| --- | --- | --- | --- | --- | --- | --- | --- |
| **1** | **I have felt cheerful and in good spirits** | 5 | 4 | 3 | 2 | 1 | 0 |
| **2** | **I have felt calm and relaxed** | 5 | 4 | 3 | 2 | 1 | 0 |
| **3** | **I have felt active and vigorous** | 5 | 4 | 3 | 2 | 1 | 0 |
| **4** | **I woke up feeling fresh and rested** | 5 | 4 | 3 | 2 | 1 | 0 |
| **5** | **My daily life has been filled with things that interest me** | 5 | 4 | 3 | 2 | 1 | 0 |
