# Supplementary material for: Subjective wellbeing among rheumatic heart disease patients at Tikur Anbessa Specialized Hospital, Addis Ababa, Ethiopia: observational cross-sectional study
Source: BMC Health Serv Res. 2021 Dec 19;21:1354. doi: 10.1186/s12913-021-07378-0 (PMC8684619; doi:10.1186/s12913-021-07378-0)
Supplement: Supplementary file 2 — Additional file 2. [file 12913_2021_7378_MOESM2_ESM.pdf]

Subjective Wellbeing among Rheumatic Heart Disease Patients at Tikur Anbessa Specialized Hospital, Addis Ababa, Ethiopia: Observational cross-sectional study

Patient's socio demography and clinical data

1. Code number _______
2. Age __________
3. Sex □ Female □ Male
4. Address _______________

   1. AA 2. Oromia 3. Amhara 4. SSNPR 5. Others _________

5. Distance of residence from Addis Ababa?

   A. < 100 Kms B. 100-200Kms 3.200-300Kms 4. >300kms

6. Educational level __________

   1. Illiterate 2. Can read and write 3. Primary education (attended grades 1-8)
   4. Secondary education (attended grades 9-12)
   5. College level education (certificate/diploma/1st degree/Master/MD/PhD)

7. Marital status (**For 18+ years of age**), **(If the subject is <18years please go to Q. 9 or if married note the response)**

   1. Married 2. Divorced 3. Widow 4. Separated 5. Cohabitating 6. Single

8. If married, how many kids do you have?
   A. Single child B. Two C. Three D. four and above
9. Occupation?
   A. Student B. Government employee C. Self-employed D. Farmer E. house wife F. others, specify___
10. Monthly family income?
    A. <1000-birr B) 1000-2999birr C) 3000-4999-birr D) 5000-10,000 birr
    E. > 10,000 birr
11. Age at the diagnosis of RHD______________ yrs
12. Severity of valvular lesions? (Pls use the higher grade if different and multiple severity grades are noted)

    A. Mild B. Moderate C. Severe

13. Are there any other comorbidities?

    ☐NO ☐Yes ………specify __________________________

14. NYHA functional class of the patient __________

    A. I B.II C.III D.IV

15. Any current or past history of RHD associated complications? (More than one response is accepted)

    A. Heart failure B. Stroke C. Myocardial Infarction D. Infective Endocarditis E. Rheumatic recurrence F. others, pls specify___________

16. Types of drugs? (More than one response is accepted)

    A. Diuretics B. Benzathine Penicillin C. Digoxin D. Aspirin/Warfarin E. others, pls specify___

17. How do you assess your adherence to drug therapy and follow up?

    A. Good B. Poor C. satisfactory

18. From where do you get any psychosocial support? (More than one response is accepted)

    A. Family B. Friends C. Religious leaders D. others

19. Any history of hospital admission in the last one year? (if response is no go to Q. 22)

    A. Yes B. no

20. Number of hospital admission (in the last one year) ________________

    1. None 2.1-2 3.3-4 4. ≥5

21. Major reason for hospitalization?

    A. Pneumonia B. Heart failure C. Stroke D. Infective Endocarditis F. others, specify____

22. Surgery or intervention done? ☐YES ☐NO, if yes pls specify timing and intervention_________

23. Nutritional status: for children and adults based on the World Health organization growth curves (Weight for height) or BMI

    A. Normal B. Mild wasting B. Moderate Wasting C. Severe wasting
